# Calcitriol enhances Doxorubicin‐induced apoptosis in papillary thyroid carcinoma cells via regulating VDR/PTPN2/p‐STAT3 pathway

**DOI:** 10.1111/jcmm.15224

**Published:** 2020-04-13

**Authors:** Ting Zhang, Liang He, Zhihong Wang, Wenwu Dong, Wei Sun, Yuan Qin, Ping Zhang, Hao Zhang

**Affiliations:** ^1^ Department of Thyroid Surgery The First Hospital of China Medical University Shenyang China

**Keywords:** apoptosis, Calcitriol, papillary thyroid carcinoma, vitamin D receptor

## Abstract

There is increasing evidence that vitamin D deficiency is the risk factor for multiple diseases, such as immune disorder, cardiovascular disease and cancer. Calcitriol is the active form of vitamin D with beneficial effects on anti‐cancer by binding vitamin D receptor (VDR). The primary aim of this study was to investigate the role of Calcitriol on papillary thyroid carcinoma (PTC) and explore the possible mechanism. We found nuclear VDR expression in PTC samples was negatively correlated with STAT3 hyperphosphorylation that indicated worse PTC clinicopathologic characteristics. Calcitriol treatment up‐regulated VDR and protein tyrosine phosphatase N 2 (PTPN2) expression, down‐regulated signal transducers and activators of transcription (STAT3) phosphorylation and thereby facilitating chemotherapy drug Doxorubicin‐induced apoptosis in PTC cell lines. However, the apoptosis‐promoting effect of Calcitriol and Doxorubicin co‐treatment was abrogated by STAT3 hyperphosphorylation, indicating suppression of STAT3 phosphorylation was essential for combined treatment of Calcitriol and Doxorubicin in PTC. Together, these results suggested that Calcitriol reinforced the sensitivity of PTC cells to Doxorubicin by regulating VDR/PTPN2/p‐STAT3 signalling pathway.

## 1 INTRODUCTION

Papillary thyroid carcinoma (PTC) is one of the most common thyroid malignancies, which is characterized by polycentric occurrence in the thyroid gland and constant cervical lymphatic metastasis.^1^ With an average prevalence of 2‐3/100 000 inhabitants, the incidence of PTC has gradually increased in recent decades.^2^ It was estimated that nearly 64 300 PTC patients were newly diagnosed in the United States in 2016.^3^, ^4^ Surgical excision, l‐thyroxine replacement therapy and adjuvant radioactive iodine (RAI) therapy are the approved treatment targeting PTC. In consideration of the tractability and the excellent survival outcome (5‐year survival rate of 90%‐95%), PTC is generally categorized as the ‘good cancer’.^5^ However, it is also reported that approximately 10%‐15% PTC patients exhibit local recurrence and metastasis even received adequate treatment.^6^, ^7^ Unfortunately, a small percentage of PTC patients are insensitive to RAI therapy or miss the opportunity for surgery, their prognosis is poor, and the 10‐year survival rate sharply drops to 40%.^8^ Therefore, it is urgent to investigate the mechanism underlying PTC for developing a more efficient clinical treatment strategy.

Recently, considerable epidemiological reports indicate that vitamin D deficiency is positively correlated with the occurrence of multiple malignancy, such as head and neck squamous cell carcinoma, prostate cancer and thyroid carcinoma.^9^, ^10^, ^11^ The circulating form of vitamin D, serum 25‐hydroxyvitamin D (25(OH)D), is used as an indirect indicator of vitamin D levels in patients, which is categorized as deficient (<20 ng/mL), insufficient (21‐29 ng/mL) and sufficient.^12^, ^13^ Although vitamin D deficiency is common in the general public, lower serum 25(OH)D levels are related to the development and severe prognosis of certain cancers. A meta‐analysis involving 2643 patients with haematological cancer has revealed that lower circulating levels of 25(OH)D were significantly related to the shrunken overall survival and the relapse‐free survival.^14^ Moreover, vitamin D3 and its analogues possess promising anti‐cancer effect in lung and breast carcinoma model systems.^15^, ^16^ Previous studies support that the active form of vitamin D3, 1,25(OH)2D3 (Calcitriol) achieves the efficient anti‐cancer effect in various tumour cells by exerting its anti‐proliferative, pro‐differentiating and pro‐apoptotic functions.^17^ Whereas the role of Calcitriol on PTC is less well known, the underlying mechanism is seldom investigated. Hence, we explored the effects of Calcitriol on PTC to deal with this issue.

Vitamin D receptor (VDR) is an essential part for the anti‐cancer effect of Calcitriol.^18^ Cytoplasmic VDRs bind to Calcitriol, emerge conformational change, form heterodimers with the binding partner retinoid X receptor (RXR) and thereby translocating to the nucleus to promote or suppress the transcription of target genes.^19^ VDR is distributed in the epithelial cells of normal and malignant thyroid; moreover, VDR mRNA levels are observably increased in PTC tissues compared with the normal thyroid.^20^ It is also reported that elevated VDR protein expressions present in typical and tall‐cell variant PTC tissues and lateral neck metastatic nodes; besides, VDR mRNA overexpression also results in poor prognostic features, including lateral cervical node metastasis, advanced stage and shorten recurrence‐free survival.^21^ The above evidence suggests VDR pathway dysfunction may involve in the pathogenesis of PTC. Hence, elaborating the molecular mechanism of VDR in PTC is essential. Protein tyrosine phosphatase N 2 (PTPN2), the downstream molecule of VDR, is believed to regulate the immune process and inflammatory reaction.^22^ Moreover, PTPN2 gene loss is associated with increased tumour cell growth and used as predictive markers of endocrine resistance in breast cancer.^23^ PTPN2 expression influences the activity of several phosphorylated proteins, including insulin receptor, Janus kinase (JAK) and signal transducers and activators of transcription 1 and 3 (STAT1 and STAT3).^24^ Particularly, widely known for its role in proliferation, apoptosis, angiogenesis and invasion of cancer cells, constitutively activated JAK/STAT3 pathway accelerates tumour progression in diverse cancer types.^25^, ^26^ Based on the above evidence, we preliminarily speculated that Calcitriol administration might be a promising adjuvant treatment for PTC. Therefore, we firstly investigated the relationship between VDR and p‐STAT3 in the malignant thyroid tissues of PTC patients. Besides, we found that Calcitriol augmented Doxorubicin‐induced apoptosis via VDR/PTPN2/p‐STAT3 signalling pathway in PTC cells.

## 2 MATERIALS AND METHODS

### 2.1 Patient samples

A total of 80 PTC patients (69 females and 11 males, mean age 45.25 years), who had undergone thyroidectomy in the First Affiliated Hospital of China Medical University, were recruited from December 2014 to April 2016. The collected patients had not exposed to multivitamins or vitamin D supplements within the 12 months. After microdissection, both thyroid carcinoma tissues and normal thyroid tissues (located more than 2 cm away from the tumour margins on the same or the opposite thyroid lobe) were collected from each PTC patient. Sample collection was implemented after the participants signed the informed consent, and all the procedures were in accordance with the Declaration of Helsinki. All the protocols were approved by the Ethics Committee of the First Affiliated Hospital of China Medical University.

### 2.2 Immunohistochemical analysis

The removed thyroid tissues were embedded in paraffin, sectioned by a rotary microtome (4 μm thick) and then baked at 60°C for 2 hours. By the method of immunohistochemistry, the samples were deparaffinized with xylene, rehydrated with graded ethanol solutions and then incubated with 3% hydrogen peroxide for 15 minutes. Afterwards, the samples were treated with primary antibodies (rabbit anti‐VDR and rabbit anti‐p‐STAT3) (Abcam) overnight at 4°C and the secondary antibody for another 15 minutes at 37°C. After washing three times in phosphate buffer (0.1 mol/L), the sections were stained by diaminobenzidine slides and counterstained with haematoxylin. Finally, the target protein was observed under a light microscopy in five random fields at 400× magnification.

### 2.3 Cell culture

Human PTC cell line K1 was obtained from the European Collection of Cell Cultures, and human PTC cell line IHH4 was gifted by Haixia Guan from the First Affiliated Hospital of China Medical University. IHH‐4 cells were established from a 75‐year‐old man with PTC, and K1 cells were derived from a metastasis of a well‐differentiated PTC and reported on mutation in BRAF (V600E). Both of them have been confirmed to be thyroid cancer cell lines that represent PTC.^27^, ^28^ The K1 and IHH4 cells were cultured as previously described.^29^ The confluent K1 and IHH4 cells were incubated with 100 nmol/L Calcitriol (Selleck) that was dissolved in DMSO (Sigma) for 24 hours before the subsequent study.

### 2.4 Transfection

When the K1 and IHH4 cells reached 20%‐30% confluency, the cells were transfected with VDR or PTPN2‐specific small interfering RNA (siRNA) (GenePharma) according to the manufacturer's instructions. After the transfection, the K1 and IHH4 cells were incubated in fresh medium for 24 hours and then exposed to 100 nmol/L Calcitriol for another 24 hours.

### 2.5 Transduction

P‐STAT3 expression lentivirus and negative control lentivirus were purchased from BioVector Biological Technology Co., Ltd. and transduced into the K1 and IHH4 cells according to the manufacturer's introduction. After the transduction, the K1 and IHH4 cells were pre‐treated with 100 nmol/L Calcitriol for 48 hours. Doxorubicin (Selleck) with a final concentration of 100 nmol/L was added to the medium; afterwards, the K1 and IHH4 cells were cultured for another 24 hours before harvest.

### 2.6 Cell treatment and proliferation

Cell Counting Kit‐8 (CCK‐8) assay (Dojindo) was utilized to detect the viability of K1 and IHH4 cells mentioned above. According to the protocols of the manufacturer, the absorbance at 450 nm was read by a microplate reader (Tecan).

### 2.7 Cell apoptosis analysis

Aforementioned K1 and IHH4 cells were stained with Annexin V‐FITC Apoptosis Detection Kit (BD Biosciences) according to the manufacturer's instructions, and the apoptotic cells were detected by flow cytometry analysis.

### 2.8 Real‐time polymerase chain reaction (RT‐PCR) analysis

The total RNA of K1 and IHH4 cells was extracted by TRIzol reagent (TAKARA). According to the manufacturer's instructions, the extracted RNA was transcribed to the corresponding cDNA with Prime Script RT Master Mix (TAKARA) and the primers (GeneChem). Relative quantification of target gene was normalized by GAPDH levels and calculated using the double standard curve method. The primers for RT‐PCR were shown as follows:

**Table:** 

SOCS3	Forward primer: 5′‐GAGGCTGGAGGTCATTGGAGAGG‐3′
	Reverse primer: 5′‐AGGTAATTCCATCGCTGCTACATTCC‐3′
SOCS5	Forward primer: 5′‐TCTGCCGTGCAGTAATCTGT‐3′
	Reverse primer: 5′‐GCCTTGACTGGTTCTCGTTC‐3′
PTPN2	Forward primer: 5′‐GAAGAGTTGGATACTCAGCGTC‐3′
	Reverse primer: 5′‐TGCAGTTTAACACGACTGTGAT‐3′
PIAS	Forward primer: 5′‐CTGGGCGAATTAAAGCACATGG‐3′
	Reverse primer: 5′‐AAAGCGTCGTCGGTAAAGCTC‐3′
VDR	Forward primer: 5′‐GGGGTCTCAGGATAGGGACT‐3′
	Reverse primer: 5′‐CCTCAGTGCCCCTTAGTGTC‐3′
Bcl‐2	Forward primer: 5′‐CGACTTTGCAGAGATGTCCA‐3′
	Reverse primer: 5′‐ATGCCGGTTCAGGTACTCAG‐3′
Bcl‐xl	Forward primer: 5′‐GTAGTGAATGAACTCTTTCGGGATGG‐3′
	Reverse primer: 5′‐AGCCACAGTCATGCCCGTCAGG‐3′

### 2.9 Western blotting analysis

The total and nuclear protein were, respectively, extracted from the aforementioned K1 and IHH4 cells as required. The protein concentration was quantified by the BCA Protein Assay Kit (KeyGEN BioTECH). Equal amount of protein was separated by 10% or 12% SDS‐PAGE and transferred onto PVDF membranes (Millipore) that were subsequently blocked by 5% bovine serum albumin (BSA) for 2 hours at room temperature. Then, the PVDF membranes were incubated with respective primary antibodies at 4°C overnight and horseradish peroxidase (HRP) labelled secondary antibody for an additional 2 hours at 37°C. Finally, chemiluminescence (ECL) kit (Millipore) was used to visualize antibody activity that was detected by Molecular Imager Gel Doc XR System (Bio‐Rad). The mean intensity of protein bands was quantified with Image‐Pro Plus 6. The primary antibodies were as follows: rabbit anti‐VDR, anti‐PTPN2, anti‐p‐STAT3, anti‐STAT3, anti‐cleaved caspase‐3, anti‐Bcl‐xl (Abcam), anti‐GAPDH and anti‐PCNA (Cell Signaling Technology).

### 2.10 Statistical analysis

Data were presented as the means ± standard derivations (SD) and analysed by Student's *t* test or one‐way analysis of variance (ANOVA) followed by Tukey's multiple comparison test. Chi‐squared test was used to analyse the clinical data. SPSS v21.0 and Prism 5.0 were applied for data analysis. *P* < .05 was considered statistically significant.

## 3 RESULTS

### 3.1 Nuclear VDR expression is negatively related to STAT3 hyperphosphorylation in PTC patients

**Figure 1:**
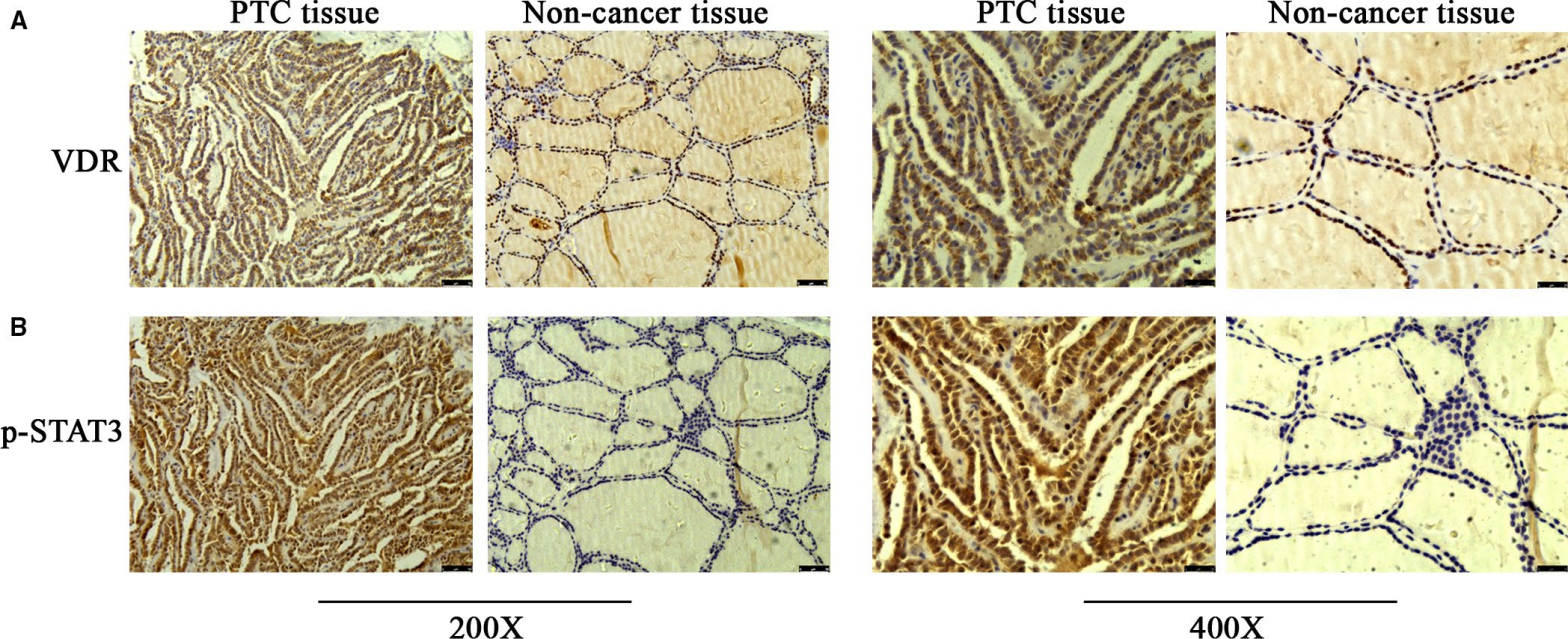
Nuclear VDR expression is negatively related to STAT3 hyperphosphorylation in PTC patients. A, The expressions of VDR were examined by immunohistochemical staining in PTC and non‐cancer tissues of patients at 200× and 400× magnification. B, The expressions of p‐STAT3 were examined by immunohistochemical staining in PTC and non‐cancer tissues of patients at 200× and 400× magnification. Scale bar 50 or 25 μm

Firstly, we examined the expression of VDR in PTC patients using immunohistochemical staining. As shown in Figure 1A, significant difference on VDR expression could be obtained between the PTC tissues and the non‐cancer tissues. Compared with the non‐cancer tissues, VDR is abundantly expressed in the PTC tissues, which is generally concentrated in cytoplasm (45/80, 56.2%). Positive immunohistochemical staining targeting p‐STAT3 was diffusely located in cytoplasm and nuclei of PTC and normal thyroid tissues, while the p‐STAT3 levels in PTC samples (51/80, 63.8%) were observably higher when compared with those in non‐cancerous tissues (21/80, 26.2%, *P* < .05, Figure 1B) (Table 1). As described in Table 2, tumour size and lymph node metastasis were positively correlated with STAT3 hyperphosphorylation; however, there was no explicit relationship between positive p‐STAT3 staining and other clinicopathologic characteristics, such as age, sex, tumour node metastasis stage and recurrence risk stratification. In addition, there is a negative correlation between p‐STAT3 levels and nuclear VDR expression in PTC tissues. Therefore, we hypothesized that activating VDR, which in turn inhibited STAT3 hyperphosphorylation, might be a potential therapeutic strategy for PTC.

**Table 1:** Expression of p‐STAT3 between PTC and non‐cancerous tissue

	p‐STAT3		*P* value^a^
	+	−	
PTC	51	29	.000*
Non‐cancerous tissue	21	59	

^a^ Chi‐square test.

* *P* < .05.

**Table 2:** Relationship between clinical manifestations and p‐STAT3 levels in PTC patients

Manifestation	p‐STAT3		*P* value^a^
	Positive	Negative	
Age			
≥45	25	16	.597
<45	26	13	
Gender			
Male	6	5	.494
Female	45	24	
Tumour size			
≤1	24	22	.012*
>1	27	7	
T‐stage			
Ⅰ + Ⅱ	38	23	.628
Ⅲ + Ⅳ	13	6	
RRS			
Low	29	17	.878
Middle + high	22	12	
LNM			
No	23	20	.039*
Yes	28	9	
VDR			
Positive	10	16	.001*
Negative	41	13	

Abbreviations: LNM, lymph node metastasis; RRS, recurrence risk stratification; T‐stage, tumour node metastasis stage; VDR, vitamin D receptor expression.

^a^ Chi‐square test.

* *P* < .05.

### 3.2 Calcitriol treatment promotes VDR and PTPN2 expression in PTC cells

**Figure 2:**
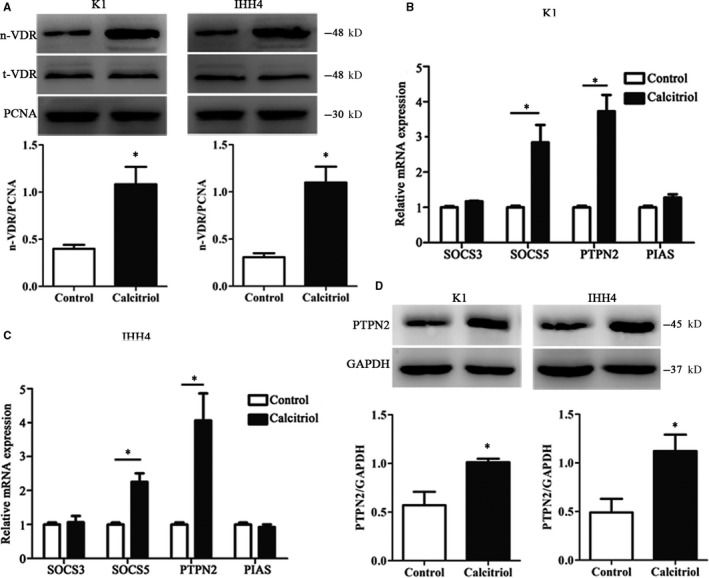
Calcitriol treatment promotes VDR and PTPN2 expression in PTC cell lines. A, The protein expressions of nuclear VDR were evaluated by Western blotting in PTC cell lines. B, The mRNA expressions of SOCS3, SOCS5, PTPN2 and PIAS were evaluated by RT‐PCR in K1 cells. C, The mRNA expressions of SOCS3, SOCS5, PTPN2 and PIAS were evaluated by RT‐PCR in IHH4 cells. D, The protein expressions of nuclear PTPN2 were evaluated by Western blotting in PTC cell lines. Data were presented as the means ± SD and analysed by Student's *t* test or one‐way analysis of variance (ANOVA) followed by Tukey's multiple comparison test (n = 3), *P < .05 vs control

To investigate the effects of VDR in the PTC pathogenesis, we treated PTC cells with the active form of vitamin D3 Calcitriol for 24 hours. As shown in Figure 2A, the nuclear expression of VDR was markedly increased with Calcitriol incubation in K1 and IHH4 cells (*P* < .05). Afterwards, the possible downstream signalling molecules of VDR were screened. As described in Figure 2B,C, PTPN2 mRNA changes were most obvious among multiple molecules after Calcitriol treatment (*P* < .05). In addition, consistent with the mRNA trend after Calcitriol stimulation, the protein levels of PTPN2 were also significantly increased in K1 and IHH4 cells (*P* < .05). The data of this section demonstrated that VDR activation might involve in the regulation of PTPN2 expression.

### 3.3 VDR knock‐down cripples the effects of Calcitriol on PTPN2 expression and STAT3 phosphorylation in PTC cells

**Figure 3:**
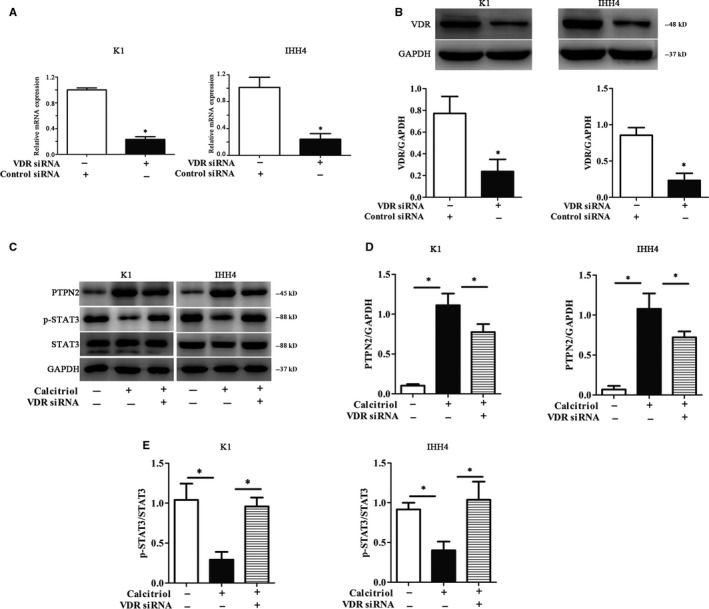
Vitamin D receptor (VDR) knock‐down cripples the effects of Calcitriol on PTPN2 expression and STAT3 phosphorylation in PTC cells. A, The mRNA expressions of VDR were evaluated by RT‐PCR in PTC cell lines. B, The protein expressions of VDR were evaluated by Western blotting in PTC cell lines. C, Representative Western blotting for PTPN2 and p‐STAT3 in PTC cell lines. D, The relative protein contents of PTPN2 in PTC cell lines. E, The relative protein contents of p‐STAT3 in PTC cell lines. The protein expressions of nuclear PTPN2 were evaluated by Western blotting in PTC cell lines. Data were presented as the means ± SD and analysed by Student's *t *test or one‐way analysis of variance (ANOVA) followed by Tukey's multiple comparison test (n = 3), *P < .05 vs control or the indicated group

To testify the effects of VDR activation in PTC cell lines, we firstly silenced VDR using specific VDR siRNA. As the data shown in Figure 3A,B, both the mRNA and protein levels of VDR were dramatically decreased after VDR knock‐down in K1 and IHH4 cells (*P* < .05). Additionally, the elevated expression of PTPN2 caused by Calcitriol treatment could be offset by VDR silence (*P* < .05, Figure 3C,D). Since STAT3 phosphorylation is widely known for its role in proliferation, differentiation and apoptosis of various tumour cells, here, we also observed the phosphorylation levels of STAT3 in K1 and IHH4 cells. As shown in Figure 3C,E, Calcitriol treatment significantly inhibited STAT3 phosphorylation (*P* < .05), which could be overturned by VDR knock‐down. VDR expression did not affect other PTPNs and STAT1 and 5 that were evaluated by RT‐PCR (*P* > .05, Figure S1). The results proved that VDR expression involved in the regulation of PTPN2 expression as well as the downstream proteins.

### 3.4 PTPN2 knock‐down diminishes the inhibitory effects of Calcitriol on STAT3 phosphorylation

**Figure 4:**
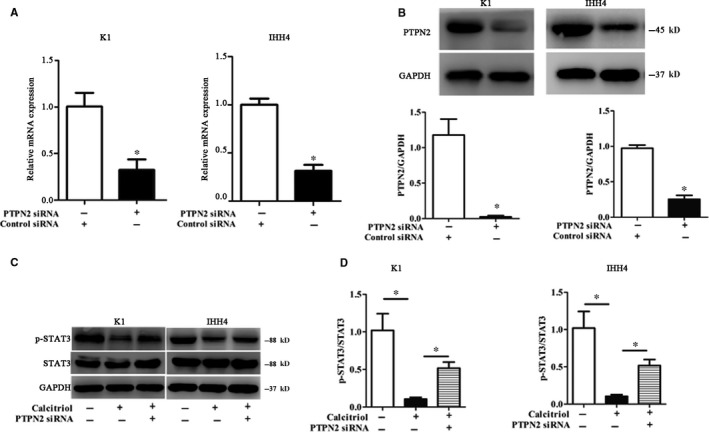
PTPN2 knock‐down diminishes the inhibitory effect of Calcitriol on STAT3 phosphorylation. A, The mRNA expressions of PTPN2 were evaluated by RT‐PCR in PTC cell lines. B, The protein expressions of PTPN2 were evaluated by Western blotting in PTC cell lines. C, Representative Western blotting for p‐STAT3 in PTC cell lines. D, The relative protein contents of p‐STAT3 in PTC cell lines. The protein expressions of nuclear PTPN2 were evaluated by Western blotting in PTC cell lines. Data were presented as the means ± SD and analysed by Student's *t *test or one‐way analysis of variance (ANOVA) followed by Tukey's multiple comparison test (n = 3), *P < .05 vs control or the indicated group

In the subsequent trials, to further verify the regulating effects of PTPN2 on STAT3 phosphorylation in PTC cells, PTPN2 was silenced utilizing PTPN2‐specific siRNA. Similar to the previous results, both the mRNA and protein levels of PTPN2 were markedly decreased with PTPN2 siRNA treatment (*P* < .05, Figure 4A,B). Moreover, the phosphorylation levels of STAT3 significantly increased with PTPN2 silence (*P* < .05, Figure 4C,D). These results suggested that PTPN2 involved in the regulation of STAT3 activity.

### 3.5 Calcitriol potentiates Doxorubicin‐induced apoptosis in PTC cell lines

**Figure 5:**
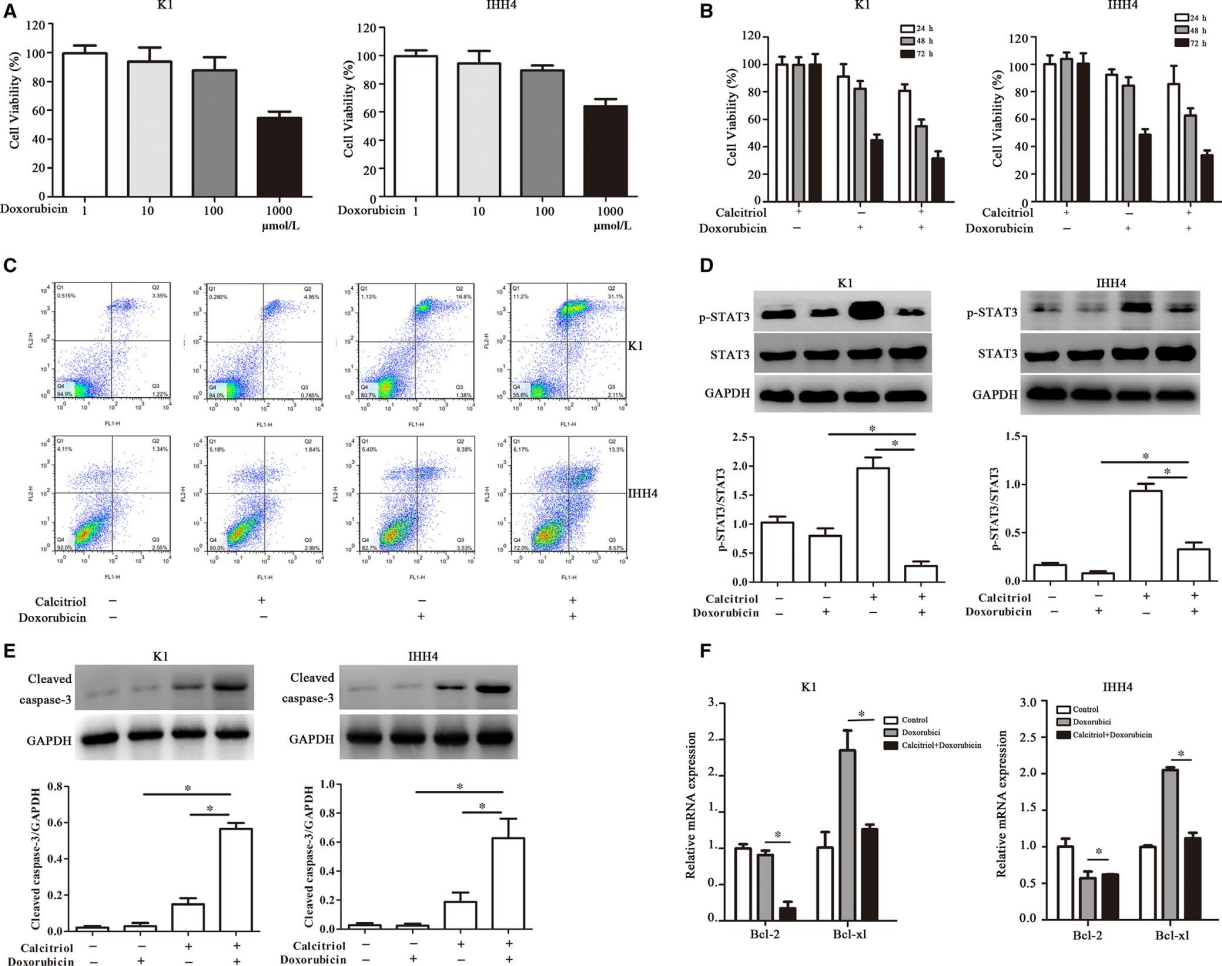
Calcitriol potentiates Doxorubicin‐induced apoptosis in PTC cell lines. A, Cell viability with doxorubicin treatment at different concentrations for 24 h. B, Cell viability with Calcitriol and doxorubicin co‐treatment for indicated time. C, Flow cytometry analysis was used to detect the apoptosis in PTC cell lines. D, The protein expressions of p‐STAT3 after Calcitriol and doxorubicin co‐treatment were evaluated by Western blotting in PTC cell lines. E, The protein expressions of cleaved caspase‐3 after Calcitriol and doxorubicin co‐treatment were evaluated by Western blotting in PTC cell lines. F, The mRNA expressions of Bcl‐2 and Bcl‐xl after Calcitriol and doxorubicin co‐treatment were evaluated by RT‐PCR in PTC cell lines. The protein expressions of nuclear PTPN2 were evaluated by Western blotting in PTC cell lines. Data were presented as the means ± SD and analysed by Student's *t* test or one‐way analysis of variance (ANOVA) followed by Tukey's multiple comparison test (n = 3), *P < .05 vs control or the indicated group

Doxorubicin is the recognized chemotherapy drug for thyroid cancer. The IC50 of Doxorubicin in IHH‐4 and K1 cells were 1102 and 1040 nmol/L, respectively. However, Doxorubicin promotes STAT3 phosphorylation, which diminishes its pro‐apoptotic effect and enhances doxorubicin resistance. In the CCK‐8 assay, Doxorubicin inhibited PTC cell viability by a dose‐dependent manner (Figure 5A). As shown in Figure 5B, pre‐treatment of Calcitriol for 48 hours significantly enhanced the inhibitory effects of Doxorubicin on PTC cell viability. In addition, Doxorubicin‐induced PTC cell apoptosis was significantly increased after Calcitriol pre‐treatment for 48 hours, which was accompanied by reduced STAT3 phosphorylation, increased cleaved caspase‐3 expression at protein levels and decreased Bcl‐2 and Bcl‐xl expression at mRNA levels (Figure 5C‐F). We detected the effect of the combination of drugs on the cell cycle by flow cytometry, and we found that the combination of drugs could not change the cell cycle (Figure S2).

**Figure 6:**
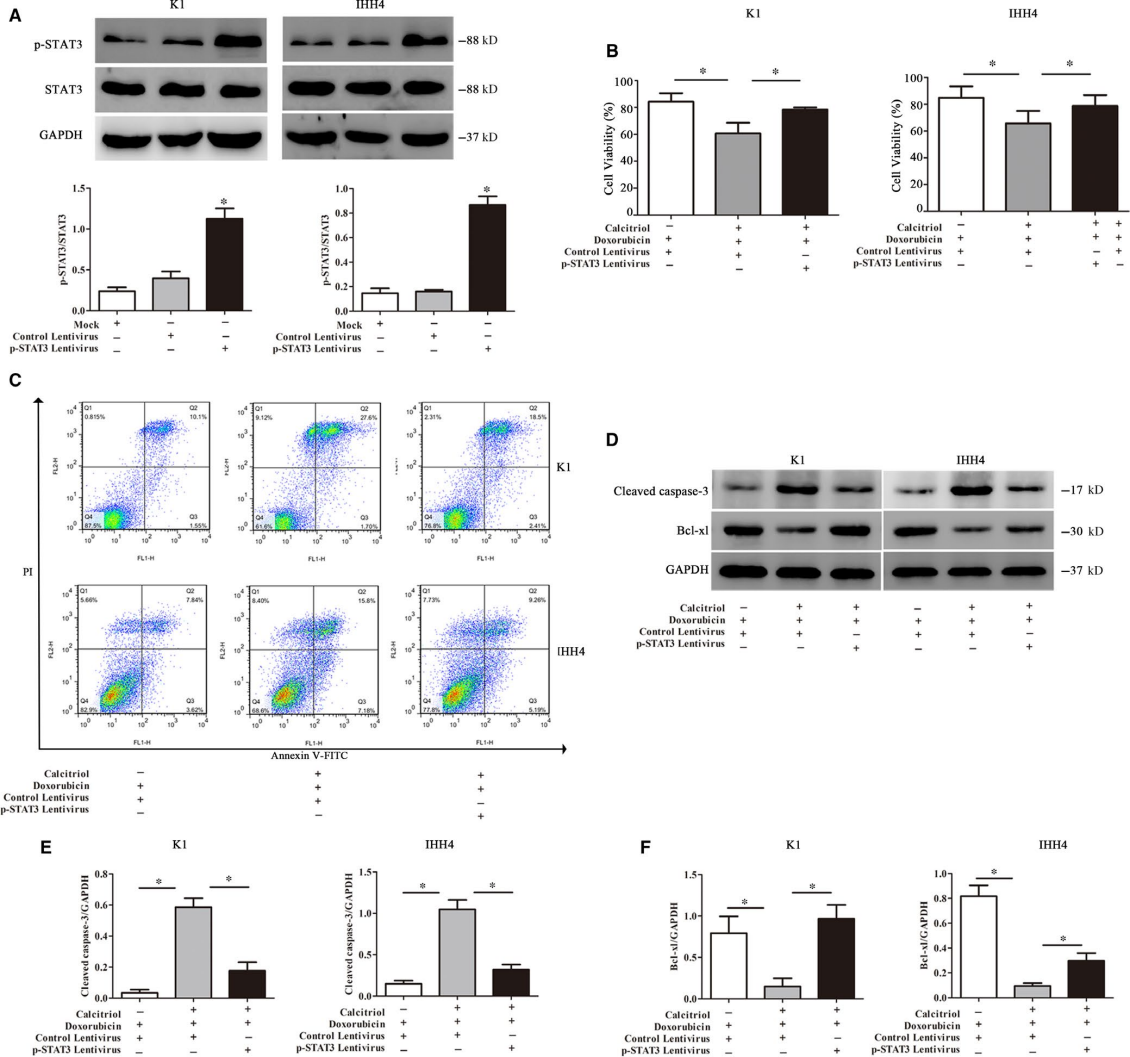
p‐STAT3 overexpression offsets the potentiation of Calcitriol on Doxorubicin‐induced apoptosis. A, The protein expressions of p‐STAT3 after p‐STAT3 expression lentivirus transduction in PTC cell lines. B, Cell viability of the PTC cell lines after p‐STAT3 expression lentivirus transduction. C, Flow cytometry analysis was used to detect the apoptosis in PTC cell lines after p‐STAT3 expression lentivirus transduction. D, Representative Western blotting for cleaved caspase‐3 and Bcl‐xl in PTC cell lines. E, The relative protein contents of cleaved caspase‐3 in PTC cell lines. F, The relative protein contents of Bcl‐xl in PTC cell lines. The protein expressions of nuclear PTPN2 were evaluated by Western blotting in PTC cell lines. Data were presented as the means ± SD and analysed by Student's *t* test or one‐way analysis of variance (ANOVA) followed by Tukey's multiple comparison test (n = 3), *P < .05 vs control or the indicated group

In order to verify the underlying mechanism, the K1 cells and IHH4 cells were transduced with p‐STAT3 expression lentivirus. p‐STAT3 levels were significantly increased in p‐STAT3 expression lentivirus‐transduced K1 cells and IHH4 cells (*P* < .05, Figure 6A), indicating p‐STAT3 overexpression using a lentivirus‐mediated system was achievable. As described in Figure 6B,C, the combination of Calcitriol and Doxorubicin resulted in decreased cell viability and increased apoptosis in PTC cells, which could be abrogated by p‐STAT3 overexpression (*P* < .05). Equally, decreased cleaved caspase‐3 expression at protein levels and increased Bcl‐xl expression at mRNA levels were also observed after p‐STAT3 overexpression (*P* < .05, Figure 6D‐F). These results suggested that Calcitriol pre‐treatment enhanced Doxorubicin sensitivity in PTC cell lines via inhibiting Doxorubicin‐related STAT3 hyperphosphorylation.

## 4 DISCUSSION

In the present study, we explored the relationship between VDR expression and STAT3 phosphorylation in PTC patients, as well as investigated the adjuvant anti‐cancer activity of Calcitriol and its potential mechanisms. We found nuclear VDR expression was negatively correlated with STAT3 hyperphosphorylation that predicted the poor clinical manifestations in PTC patients. Our work showed that Calcitriol pre‐treatment enhanced Doxorubicin‐induced apoptosis in PTC cells, which was accompanied by increased nuclear VDR expression, promoted PTPN2 activity and attenuated STAT3 phosphorylation. Thus, our present result manifested that Calcitriol contributed to the chemotherapy sensitivity of PTC cells via regulating VDR/PTPN2/p‐STAT3 signalling.

Accumulating evidence has suggested that lower circulating vitamin D levels are related to a higher incidence of developing various cancers, probably through the genomic effects regulated by VDR.^30^ Calcitriol, vitamin D3 with biological activity, involves in the transcription of many genes modulating apoptosis, proliferation and differentiation. Moreover, it is noteworthy that abundant VDR expression is found not only in normal thyroid follicular cells, but also in PTC cells.^4^ Such biological features of VDR distribution imply the therapeutic potential of Calcitriol and its analogues against PTC. Additionally, STAT3 is an important transcription factor that modulates gene expression associated with cell cycle and survival.^31^ Once activated, STAT3 promotes the gene transcriptive processes referring to anti‐apoptosis, angiogenesis and invasion/migration.^32^ Hence, we investigated VDR expression and STAT3 phosphorylation in PTC tissues and analysed their relationship. In our study, we found the negative relationship between VDR and p‐STAT3 protein levels in the nuclei of PTC tissues. Furthermore, the p‐STAT3 level was significantly different in tumour size and lymph node metastasis, rather than other clinical characteristics, including age, sex, tumour node metastasis stage and recurrence risk stratification. Notably, it can be preliminarily predicted that VDR expression and STAT3 dephosphorylation by Calcitriol administration might be the potential signalling in the process of PTC treatment; nevertheless, the accurate mechanism is left unknown.

Despite of participating in calcium homeostasis and bone metabolism, vitamin D possesses protective function in various pathophysiological conditions, including immune disorder, cardiovascular disease and cancer.^33^, ^34^ Given that the salutary effects of vitamin D are mediated by VDR expression, which is the single nuclear high‐affinity receptor binding the active form of vitamin D3 (Calcitriol). Namely, Calcitriol binds cytoplasmic VDR, induces its conformational change and heterodimerization with RXR and thereby transferring into the nucleus to function as a transcription factor regulating proliferation, apoptosis and differentiation.^35^ Although multiple signalling transduction cascades at the molecular level affected the above process, we only explored the pathway related to VDR expression. As an important molecule of the downstream of VDR pathway, intracellular tyrosine‐specific phosphatase PTPN2 has been found to dephosphorylate and inactivate STAT3 by Shuai's laboratory in 2002.^36^, ^37^, ^38^ Since PTPN2 regulates the transcription of several oncoproteins, PTPN2 has been defined as a tumour suppressor. PTPN2 deficiency observably increased the proliferation ability of T cell receptor‐dependent naive T cells in lymphopenic hosts.^37^ More importantly, PTPN2 expression deficiency has been shown to contribute to the rapid development and poor outcome in patients with breast cancer.^23^ Nonetheless, it is not clear whether VDR and its downstream molecule PTPN2 are related to PTC development. Hence, we administrated PTC cells with Calcitriol stimulation and our data showed that Calcitriol treatment enhanced nuclear VDR expression and PTPN2 activity. In accordance with the previous studies, STAT3 acts as a possible target of PTPN2 by dephosphorylation and the levels of p‐STAT3 are dramatically elevated in various cancer cell lines.^39^, ^40^ After phosphorylation, STAT3 forms homodimer itself through reciprocal binding at the SH2 domain, transfers to the nucleus, modulates target gene transcription via binding to DNA and thereafter accelerating cell growth and blocking apoptosis.^41^, ^42^ Several lines of evidence show that the activation of STAT3 is instantaneous in normal cells; however, STAT3 phosphorylation is constitutively accelerated in cancer cells.^43^ Hence, the subsequent experiments were performed to explore the effects of Calcitriol on STAT3 phosphorylation and apoptosis in PTC cells. Doxorubicin, an important member of anthracyclines, possesses anti‐tumour effects through intercalating within DNA pairs, which suppresses DNA topoisomerase II activity and results in cell cycle blockage.^44^ In consideration of the non‐tissue‐specific characteristics, Doxorubicin has been widely used in the treatment of multiple malignancy, including breast carcinoma, neuroblastoma, ovarian carcinoma and most recurrent or metastatic cancer.^45^ Besides, the standard chemotherapy regimen is the approved treatment targeting thyroid cancer. Unfortunately, the adverse effects of Doxorubicin, such as immunosuppression, hepatotoxicity and cardiotoxicity, limit its efficacy and application. Furthermore, STAT3 hyperphosphorylation is thought to be highly related to Doxorubicin resistance, suggesting additional therapeutic strategy is needed.^46^ Subsequently, improved approach to reduce Doxorubicin side‐effects and enhance Doxorubicin efficiency is worth to explore. In our study, we evaluated the effects of Calcitriol pre‐treatment on Doxorubicin‐induced apoptosis in PTC cells and our results revealed that combining Calcitriol with Doxorubicin significantly promoted PTC cell apoptosis by inhibiting STAT3 hyperphosphorylation. In addition, the pro‐apoptotic effects of Calcitriol could be diminished by p‐STAT3 overexpression. Accordingly, our study demonstrated that Calcitriol enhanced chemotherapy sensitivity to Doxorubicin via VDR/PTPN2/p‐STAT3 signalling in PTC cells.

In conclusion, Calcitriol possesses the ability to promote Doxorubicin‐induced PTC cell apoptosis via regulation of VDR/PTPN2/p‐STAT3 signalling. Although our experiment has certain limitations and further exploration is still needed, our present study indicates that combination therapy of Calcitriol and Doxorubicin is a potential treatment strategy for PTC.

## CONFLICT OF INTEREST

The authors declare that they have no conflict of interest.

## AUTHORS’ CONTRIBUTIONS

Hao Zhang provided the conception of the study and edited the final manuscript. Ting Zhang contributed significantly to manuscript design and preparation. Liang He and Zhihong Wang finished clinical data collection and literature research. Wenwu Dong and Wei Sun performed pathological examination. Yuan Qin and Ping Zhang provided clinical information.

## Supporting information

Fig S1

Fig S2

## Data Availability

Some or all data generated or used during the study are available from the corresponding author by reasonable request.
