# Supplementary figures and images for: Calcitriol enhances Doxorubicin‐induced apoptosis in papillary thyroid carcinoma cells via regulating VDR/PTPN2/p‐STAT3 pathway
Source: J Cell Mol Med. 2020 Apr 13;24(10):5629–39. doi: 10.1111/jcmm.15224 (PMC7214146; doi:10.1111/jcmm.15224)

K1

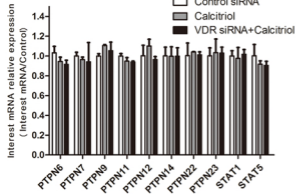

IHH4

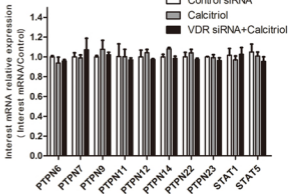

Supplement: Supplementary file 1 — Fig S1 [file JCMM-24-5629-s001.pdf]

A

K1

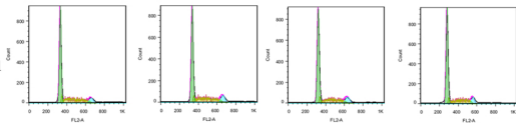

IHH4

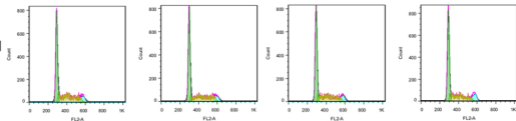

Control

Doxorubicin

Calcitriol

Doxorubicin+Calcitriol

B

K1

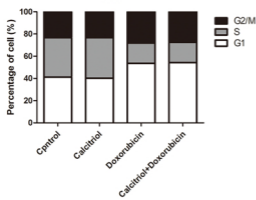

IHH4

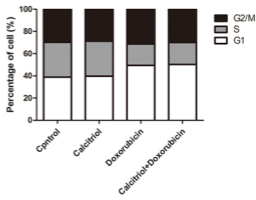

Supplement: Supplementary file 2 — Fig S2 [file JCMM-24-5629-s002.pdf]
